# Early Life Stress Effects on Glucocorticoid—BDNF Interplay in the Hippocampus

**DOI:** 10.3389/fnmol.2015.00068

**Published:** 2015-11-16

**Authors:** Nikolaos P. Daskalakis, Edo Ronald De Kloet, Rachel Yehuda, Dolores Malaspina, Thorsten M. Kranz

**Affiliations:** ^1^Traumatic Stress Studies Division and Laboratory of Molecular Neuropsychiatry, Department of Psychiatry, Icahn School of Medicine at Mount SinaiNew York, NY, USA; ^2^Mental Health Patient Care Center, James J. Peters Veterans Affairs Medical CenterBronx, NY, USA; ^3^Department of Medical Pharmacology, Leiden Academic Centre for Drug ResearchLeiden, Netherlands; ^4^Department of Endocrinology and Metabolism, Leiden University Medical Center, Leiden UniversityLeiden, Netherlands; ^5^Department of Neuroscience, Icahn School of Medicine at Mount SinaiNew York, NY, USA; ^6^Department of Psychiatry, New York University School of MedicineNew York, NY, USA; ^7^Departments of Cell Biology, Physiology and Neuroscience, and Psychiatry, Skirball Institute of Biomolecular Medicine, New York UniversityNew York, NY, USA

**Keywords:** early life stress, glucocorticoid, glucocorticoid receptor, BDNF, HPA-axis, TrkB, hippocampus

## Abstract

Early life stress (ELS) is implicated in the etiology of multiple psychiatric disorders. Important biological effects of ELS are manifested in stress-susceptible regions of the hippocampus and are partially mediated by long-term effects on glucocorticoid (GC) and/or neurotrophin signaling pathways. GC-signaling mediates the regulation of stress response to maintain homeostasis, while neurotrophin signaling plays a key role in neuronal outgrowth and is crucial for axonal guidance and synaptic integrity. The neurotrophin and GC-signaling pathways co-exist throughout the central nervous system (CNS), particularly in the hippocampus, which has high expression levels of glucocorticoid-receptors (GR) and mineralocorticoid-receptors (MR) as well as brain-derived neurotrophic factor (BDNF) and its receptor, tropomyosin-related kinase receptor B (TrkB). This review addresses the effects of ELS paradigms on GC- and BDNF-dependent mechanisms and their crosstalk in the hippocampus, including potential implications for the pathogenesis of common stress-related disorders.

## Introduction

Glucocorticoids are steroid hormones and the end product of the hypothalamus-pituitary-adrenal (HPA) axis, which regulates the stress response. GC effects are mediated by MR and GR (De Kloet et al., 1998; McEwen, 1998; de Kloet et al., 2005a);. MR and GR are abundantly expressed in hippocampus and hippocampal function is implicated in both appraisal processes and stress adaptation. Through MR, GCs influence the brain's appraisal of novel information and memory retrieval, and thereby influence behavioral coping responses (de Kloet et al., 2005a). As GC concentrations increase in response to stressors, GR are activated to promote stress adaptation, reallocation of energy resources in preparation for future events and recovery of the system (de Kloet, 2003; de Kloet et al., 2005a).

One important target of GCs is BDNF-signaling, which is a crucial contributor to the modulation of axonal guidance, synaptic plasticity and neurite outgrowth (Jeanneteau and Chao, 2013). MR, GR and the BDNF receptor, TrkB, are co-expressed in hippocampal neurons, supporting this region as the primary site of immediate interactions between the GC- and BDNF-signaling pathways (Jeanneteau et al., 2012).

In this mini-review we present the effects of ELS on GC- and BDNF-dependent mechanisms in the hippocampus using primarily evidence from ELS animal models. The most commonly used rodent and non-human primate model in this context is the maternal separation (MS) paradigms with each displaying variations in developmental age, repetition and duration (Table 1). GC- and BDNF-signaling pathways influence each other and here we propose that ELS provokes a change in their equilibrium that contributes to heightened risk for stress-related psychopathology.

**Table 1:** **Overview of rodent studies on BDNF signaling and Maternal Separation**.

**Paradigm**	**Species**	***BDNF* gene regulation**	**BDNF protein**	**HPA-axis**	**Neurogenesis and synaptic plasticity**	**Behavior**	**References**
MD (pnd 3; 24 h once)	Brown Norway rats	Month 30–32 Basal: n.c. *BDNF* mRNA in HIP +AS: ↓*BDNF* mRNA in HIP (only in MD rats with MWM impairments)					Schaaf et al., 2001
MD (pnd 7; 3 h or 6 h once)	Sprague–Dawley rats	pnd 7; 3 h after onset ↑*BDNF* exon III mRNA in HIP 6 h after onset ↓*BDNF* exon I mRNA in HIP					Nair et al., 2007
MD (pnd 9; 24 h once)	Wistar rats	pnd 9; 2, 6 or 24 h after onset n.c. *BDNF* mRNA in HIP pnd 21 n.c. *BDNF* mRNA in HIP			pnd 21 n.c. *NMDA-R mRNA* in HIP		Roceri et al., 2002
		pnd 72 ↓*BDNF* mRNA in HIP (but not further ↓ by AS)	pnd 72 ↓ BDNF protein in HIP		pnd 72 ↓*NMDA-R mRNA* in HIP		
MD (pnd 9; 24 h once)	Wistar rats	pnd 90—basal (males + females): ↓*BDNF* mRNA in HIP pnd 90 – CS (males + females): ↓*BDNF* mRNA in HIP		pnd 90—basal (males): ↓ GR in HIP pnd 90 – CS (males): ↓ GR in HIP		pnd 80–82 basal (males): ↓ NOR	Llorente et al., 2011
MD (pnd 9; 24 h once)	Wistar rats	pnd 98–112—basal: ↑*BDNF exon I, IV* mRNA in dorsal HIP (males and females) ↑*BDNF exon II, VII, IX* mRNA in dorsal HIP (males) n.c. in ventral HIP (males and females) ↑*BDNF I* mRNA in medial PFC (males) ↓*BDNF VII and VIII* mRNA in medial PFC (males) n.c. in CPu and NAc (males and females)	pnd 98–112—basal: ↓ BDNF protein in dorsal HIP (males)		pnd 98–112—basal: n.c. in CPu and NAc (males and females)	pnd 84–105—basal basal: n.c. in short-term or long-term memory spatial memory, working memory, NOR, EPM	Choy et al., 2008; Hill et al., 2014a,b
		pnd 98–112—CCORT: ↓*BDNF* mRNA in HIP (males) ↑*BDNF IX* mRNA and BDNF protein in medial PFC (males) n.c. in CPu and NAc (males and females)	pnd 98–112—CCORT: ↓ BDNF protein in ventral HIP (females)		pnd 98–112—CCORT: ↑*DR3* mRNA in medial PFC (males) ↑*DR2* mRNA in medial PFC (males) n.c. in CPu and NAc (males and females)	pnd 84–105—CCORT: ↓ short-term spatial memory (males) and learning delay in long-term spatial (males) memory, ↓ sucrose preference (females) n.c. working memory, NOR, EPM	
MD (pnd 11; 24 h once)	Sprague–Dawley (mothers) and Long Evans (fathers) hybrid rats	pnd 11; 24 h after onset: ↓*BDNF* mRNA in HIP		24 h after onset: n.c. ACTH, ↑ CORT	24 h after onset: n.c. neurogenesis in HIP ↓ neurogenesis in parietal cortex		Zhang et al., 2002
RMS (pnd 1–14; 2 h daily)	C57BL/6J (B6) and Balb/cJ (Balb/c) mice	pnd 40 n.c. *BDNF* exon IV promoter methylation (males + females) in PFC and HIP n.c. *BDNF* exon IX promoter methylation (males + females) in PFC ↑*BDNF* exon IX promoter methylation (males + females) in HIP n.c. *BDNF* exon IX promoter methylation (males + females) in PFC ↓*BDNF* mRNA (females) in HIP ↓*BDNF* mRNA (males + females) in PFC		pnd 40 ↓ GR exon 1 methylation (females) in PFC ↑ GR exon 1 methylation (males) in HIP n.c. *GR* mRNA (males+females) in HIP n.c. *GR* mRNA (males + females) in PFC		pnd 35 ↑ OF activity (males) pnd 35 ↓ sucrose preference pnd 40 ↓ social interaction (males)	Kundakovic et al., 2013
RMS (pnd 1–14; 3 h daily)	Wistar rats		pnd 104 ↓ BDNF protein in HIP (n.c. in PFC)			pnd 104 n.c. OF, n.c. NOR	Pinheiro et al., 2015
RMS (pnd 1–21; 3 h daily)	Wistar rats		pnd 56 ↓ BDNF protein in medial PFC n.c. BDNF protein in HIP n.c. BDNF protein in NAc			pnd 56 Partially impaired reversal learning performance	Xue et al., 2013
RMS (pnd 2–6; 3 h daily)	Long-Evans rats		pnd 7 MS prevented the conditioning associated ↓ BDNF protein in HIP ↓ BDNF protein in olfactory bulb			pnd 7 ↓ odor conditioning	Zimmerberg et al., 2009
RMS (pnd 2–6; 5 h daily)	B6C3Fe reeler (C57/BLJ background) mice (wild-type presented)		adult ↓ BDNF protein PFC and striatum (n.c. HIP)			adult ↓ social interaction (home cage activity n.c.)	Ognibene et al., 2008
RMS (pnd 2–14; 3 h daily)	Sprague–Dawley rats	pnd 17 basal: ↑*BDNF* mRNA in HIP and PFC (+AS no further increase) pnd 35 n.c. pnd 90 ↓*BDNF* mRNA in PFC (+CS no further increase) n.c. *BDNF* mRNA in PFC and striatum (+CS: prevented ↓ caused by CS)		pnd 1 basal CORT: n.c. +AS CORT: n.c. pnd 13 basal CORT: n.c. +AS CORT: n.c.			Roceri et al., 2004
RMS (pnd 2–14; 3 h daily)	SERT knockout Wistar rats (wild-type presented)	pnd 85-95 n.c. *BDNF* mRNA in dorsal HIP ↓*BDNF* total, 3′-UTR, exon IV mRNA in ventral HIP n.c. *BDNF* mRNA in dorsomedial PFC ↓*BDNF* total, 3′-UTR, exon IV mRNA in ventromedial PFC					Calabrese et al., 2015
RMS (pnd 2–14; 3 h daily)	Sprague–Dawley rats	pnd 14 ↑*BDNF* exon II mRNA in HIP			pnd 15 n.c. neurogenesis in HIP SGZ		Nair et al., 2007
		pnd 21 ↑*BDNF* exon IV and V mRNA in HIP			pnd 21 ↑ neurogenesis in HIP SGZ		
		pnd 60 basal: n.c. *BDNF* mRNA +AS: prevented ↓ in exon III, IV and V *BDNF* mRNA caused by AS + CS: prevented the increase in exon I and II and ↓ in exon III, IV and V *BDNF* mRNA caused by CS					
RMS (pnd 2–14; 3 h daily)	Sprague–Dawley rats		pnd 68 + JS: ↑ BDNF protein in ventral HIP (additionally MS prevented ↓ BDNF protein in dorsal HIP)	pnd 68 + JS basal: ↓ ACTH and CORT		pnd 67 + JS: n.c. EPM and OF	Faure et al., 2006, 2007
RMS (pnd 2–14; 3 h daily)	Sprague–Dawley rats	pnd 90 ↑*BDNF* mRNA in HIP			pnd 90 n.c. neurogenesis in HIP		Greisen et al., 2005
RMS (pnd 2–14; 3 h daily)	Long Evans rats		pnd 95 ↑ pro-BDNF in VTA ↓ BDNF protein in HIP, striatum ↑ BDNF protein in VTA	pnd 70–95 AS: ↑ ACTH and CORT	–	pnd 50–60 ↑ locomotor activity, ambulation and grooming ↑ acoustic startle	Lippmann et al., 2007
RMS (pnd 2–14; 3 h daily)	Sprague–Dawley rats		pnd 52 ↓ BDNF protein in dorsal HIP and ventral HIP pnd 101 ↑ BDNF protein in ventral HIP		pnd 101 n.c. MKP-1 levels in ventral HIP	pnd 65 n.c. OF activity ↑ 22 kHz vocalizations ↑ FST immobility pnd 99 ↓ EPM anxiety pnd 100 ↑ 22 kHz vocalizations ↑ FST immobility	Dimatelis et al., 2012, 2014
RMS (pnd 2–14; 3 h daily)	Sprague–Dawley rats	pnd 21 ↓ H3K9 dimethylation in HIP ↑*BDNF* exon IV mRNA in HIP ↑*BDNF* mRNA in HIP	pnd 21 ↑ BDNF protein in HIP		pnd 21 ↑ neurogenesis in HIP SGZ		Suri et al., 2013
		pnd 60 ↓ H3K9 dimethylation in HIP ↑*BDNF* exon IV mRNA in HIP ↑*BDNF* mRNA in HIP	pnd 60 ↑ BDNF protein in HIP		pnd 60 n.c. neurogenesis in HIP SGZ	pnd 60 ↑ WWM escape latency n.c. retention in WWM n.c. NOR	
		month 15 ↑ H3K9 dimethylation in HIP ↓*BDNF* exon IV mRNA n.c. *BDNF* mRNA in HIP	month 15 n.c. BDNF protein in HIP		month 15 ↓ neurogenesis in HIP SGZ	month 15 n.c. WWM escape latency ↓ retention in WWM ↓NOR	
RMS (pnd 3–12; 3 h daily)	Sprague–Dawley rats		pnd 56–70 n.c. BDNF protein in solitary tract ↑ BDNF protein in PVN ↓ BDNF protein in phrenic motor nucleus		pnd 56–70 ↑ AMPA receptor binding in solitary tract, PVN and phrenic motor nucleus	pnd 56–70 ↑ hypoxic chemoreflex	Gulemetova et al., 2013
RMS (pnd 3–15; 3 h daily)	Sprague–Dawley rats		pnd 51 ↓ BDNF protein in HIP	pnd 51 n.c. basal or AS CORT +JS: reduced the effect of JS on decreasing the basal CORT and increasing AS CORT	pnd 51 ↓ Arc in HIP		Biggio et al., 2014
RMS (pnd 10–15; 3 h daily)	Wistar rats	pnd 16 ↑*BDNF* mRNA in cerebral cortex ↑*BDNF* mRNA in cerebellum ↓*BDNF* mRNA in HIP pnd 20 ↑*BDNF* mRNA in cerebral cortex n.c. *BDNF* mRNA in cerebellum n.c. *BDNF* mRNA in HIP pnd 30 n.c. *BDNF* mRNA in cerebral cortex ↑*BDNF* mRNA in cerebellum (BDNF protein) ↑*BDNF* mRNA in HIP pnd 60 ↓*BDNF* mRNA in cerebral cortex n.c. *BDNF* mRNA in cerebellum ↑*BDNF* mRNA in HIP					Kuma et al., 2004; Lee et al., 2012; Miki et al., 2013, 2014
RMS (pnd 2–21; 3 h daily)	Wistar rats	pnd 60–75 ↓*BDNF* mRNA in HIP			pnd 60–75 ↓NCAM and *SYP* mRNA in HIP	pnd 60–75 ↓ retention in MWM	Aisa et al., 2009
RMS (pnd 2–21; 3 h daily)	Wistar rats	pnd 90 ↓*BDNF* mRNA in HIP		pnd 90 ↓ GR in HIP ↑ CORT	pnd 90 n.c. p-Akt, p-GSK3β, p-ERK1, ↓ p-ERK2 in HIP ↓*Arc* mRNA in HIP	pnd 90 n.c. OF activity ↑ FST immobility ↓ retention in MWM ↓novel object recognition	Solas et al., 2010
		month 18 ↓*BDNF* mRNA in HIP		month 18 n.c. GR in HIP n.c. CORT	month 18 n.c. p-Akt, p-GSK3β, p-ERK1, ↓ p-ERK2 in HIP ↓*Arc* mRNA in HIP	month 18 n.c. OF activity ↑ FST immobility ↓ retention in MWM ↓novel object recognition	
RMS (pnd 2–22; 3 h daily)	C57Bl/6J mice	pnd 61 + AS: ↓*BDNF* mRNA				pnd 60–61 reduced swim times in FST	MacQueen et al., 2003

*↑, increased by the applied maternal separation paradigm; ↓, decreased by the applied maternal separation paradigm; ACTH, adrenocorticotropin; AKT, v-akt murine thymoma viral oncogene homolog; AMPA, α-amino-3-hydroxy-5-methyl-4-isoxazolepropionic acid; Arc, activity-regulated cytoskeleton-associated protein; AS, acute stress; BDNF, brain derived neurotrophic factor; CCORT, chronic corticosterone treatment; CORT, corticosterone; Cpu, Caudate Putamen; CS, Chronic stress; DRD2, dopamine receptor D2; DRD3, dopamine receptor D3; ERK, Extracellular-signal-regulated kinases; FST, forced swim test; GR, glucocorticoid receptor; GSK3 β, glycogen synthase kinase 3 beta; HIP, hippocampus; MKP1, mitogen-activated protein kinase phosphatase 1; MWM, Morris Water maze; n.c., not changed by the applied maternal separation paradigm; NCAM, (Neural cell adhesion molecule); NOR, novel object recognition; OF, open field; p-, phosphorylated protein form; PFC, prefrontal cortex; pnd, postnatal day; PVN, Paraventricular nucleus of hypothalamus; RMS, repeated maternal separation; SGZ, subgranular zone; SYP, synaptophysin; VTA, ventral tegmental area*.

## HPA-axis and ELS

There is a distinct pattern of HPA-axis activity during early development first described in rodents, which maintains stable and low circulating GC levels during the stress hyporesponsive period [SHRP; postnatal days (pnd) 1–10 in mice and pnd 3–14 in rats] (Sapolsky and Meaney, 1986; de Kloet et al., 2005b). Early life experiences can disrupt the SHRP by elevating basal GC secretion and turning the HPA-axis responsive to subsequent stressors. Thus, ELS not only exerts acute effects but also impacts long-term developmental trajectories in the brain (de Kloet et al., 2005b). In humans, the SHRP occurs during the postnatal months 6–12 and adverse experiences during this period can have a long-lasting impact on the HPA-axis (Gunnar and Quevedo, 2007).

MS is an established procedure of inducing acute stress effects during early life that yields long-term effects. MS results in heightened HPA-axis responsiveness in the early postnatal period and triggers a variety of stress-related behavioral phenotypes in later life (Daskalakis et al., 2014). However, MS effects are dependent on many factors, including the duration and frequency of the separations, age of the pups, and context under which the pups experienced the separation from the dam (Rosenfeld et al., 1992; van Oers et al., 1998; Enthoven et al., 2010; Daskalakis et al., 2011). Furthermore, the long-term effects of MS depend on match or mismatch with later life context (Daskalakis et al., 2012). Studies using other ELS paradigms (variations of maternal care, limited nesting) demonstrate similar long-term effects that are mediated through GC-dependent mechanisms (Liu et al., 1997; Champagne et al., 2008; Ivy et al., 2008).

Long-lasting alterations in the HPA-axis induced by ELS in rodents have been linked to experience-dependent epigenetic modifications in regulatory regions of stress-related genes (Weaver et al., 2004; Murgatroyd et al., 2009). In humans, where early adversity is associated with adult stress-related disorders and HPA-axis dysregulation, similar epigenetic changes were reported as observed in the above-mentioned rodent studies (McGowan et al., 2009; Daskalakis and Yehuda, 2014; Ruby et al., 2015). Interestingly, epigenetic changes caused by ELS might depend on genetic predisposition (Klengel et al., 2013). Therefore, the interplay of genetic background (hit-1) with early experiences (hit-2), might create a vulnerable or resilient neuroendocrine profile which, upon adult stress exposure (hit-3), can produce an adaptive healthy or a maladaptive pathologic response (Daskalakis et al., 2013).

## BDNF signaling and ELS

ELS has consequences for structural and physiological properties of stress-sensitive brain regions and behavior. For instance, rats with a history of low maternal care displayed decreased hippocampal synaptogenesis, BDNF, long-term potentiation and memory at baseline (Liu et al., 2000). Neurotrophins are crucial mediators in the facilitation of brain connectivity, neuronal plasticity, synaptic integrity and the promotion of basal neurogenesis (Ghosh and Greenberg, 1995; Lee et al., 2002). The most abundant neurotrophin in the mammalian CNS is BDNF. It is synthesized in the endoplasmic reticulum as a pre-pro-molecule and undergoes two cleavage steps from pre-pro via pro-BDNF to its mature form, which is packaged in secretory vesicles (Pang et al., 2004; Revest et al., 2014). Upon neuronal activity, BDNF is released from the synapse and diffuses to its receptor TrkB. Upon BDNF-binding, TrkB undergoes homodimerization and autophosphorylation and thus, the activation of downstream signaling cascades involved in neuronal integrity and survival (Chao, 2003). Genetic modifications of BDNF have a crucial effect on synaptic plasticity as shown in an animal study using BDNF heterozygous (+/Met) for the Val66Met polymorphism. After 7 days of restraint stress with BDNF het and wild-type (WT) mice, BDNF het mice displayed reduced apical dendrite density in the prefrontal cortex (PFC) and in addition, impaired working memory in comparison to WT littermates (Yu et al., 2012). Moreover, BDNF influences synaptic transmission and its efficacy is influenced by this single nucleotide polymorphism (SNP) in its prodomain. In the same mouse model, the amount of NMDA receptor mediated currents in the hippocampus and the infralimbic medial PFC of BDNF Met/Met mice was significantly lower than in BDNF Val/Val mice (Ninan et al., 2010; Pattwell et al., 2012). The human BDNF gene seems to be under high selection pressure against genetic variability, since in a whole exome sequencing study of 14 schizophrenia trios and a subsequent study performing targeted exome capture in 48 sporadic schizophrenia cases, both cohorts displaying a high number of cases with childhood trauma, no novel genetic variants in the BDNF gene were observed (Kranz et al., 2015a,b).

BDNF-signaling is also influenced by ELS (Alleva and Francia, 2009). In humans, ELS can evoke significant memory impairments in adulthood (Bremner et al., 2003) in association with reduced BDNF levels (Grassi-Oliveira et al., 2008). Moreover, these associations depend on the Val66Met polymorphism (Chen et al., 2006; Elzinga et al., 2011; Molendijk et al., 2012). A similar pattern was observed for peripheral BDNF expression in young rhesus macaques. Carriers of the Met allele of the functionally ortholog polymorphism at codon 46 displayed decreased BDNF levels after maternal deprivation (Cirulli et al., 2011). Besides the combined effects of genetics and MS on BDNF expression, there are epigenetic effects associated with ELS. The relationship of MS and epigenetic regulation of BDNF has been studied extensively in rodent models (Table 1).

### Effects of ELS on BDNF mRNA and protein expression

There are too few studies to conclude on the direction of changes in BDNF expression levels in the hippocampus or other brain regions (<24 h after first maternal separation onset). One possibility is that BDNF expression decreases acutely after MS. However, pups exposed to MS also experience dietary restriction due to the absence of the mother. It has been demonstrated that dietary restriction increases BDNF expression in the hippocampus, striatum and the PFC in rats (Duan et al., 2001).

In the postweaning period and depending on the characteristics of the MS protocol, increased BDNF expression is reported more consistently. In the period between adulthood and senescence, BDNF expression is reduced, but the time of the switch depends on experimental characteristics (ELS paradigm, stress context in adulthood), sex, rodent strain and brain region of interest.

### Effects of ELS on epigenetic regulation of *BDNF*

ELS influences the methylation status of the activity-dependent *BDNF* exon IV expression. One study has shown in rats that repeated maternal separation (RMS) leads to a biphasic effect of the exon IV promoter methylation status. At P21, RMS results in lower H3K9 dimethylation of the exon IV promoter but from adolescence (2 months) into adulthood (15 months), the initially decreased dimethylation after RMS reverses into a significantly increased dimethylation. Low dimethylation status at P21 yields a high *BDNF* exon IV transcription and vice versa during adolescence and adulthood (Suri et al., 2013). In another study where the exon IV promoter methylation change was not confirmed, increased exon IX promoter methylation was reported in hippocampus in maternally separated pups (Kundakovic et al., 2013). In a different ELS paradigm, rat dams with restricted availability of nesting material resulted in reduced maternal licking and grooming behaviors toward their pups and reduced physical interaction between the dams and their offspring (Ivy et al., 2008). This paradigm mimics infant neglect as well, which entails increased basal corticosterone levels in the offspring (Rice et al., 2008). Interestingly, maltreated offspring display hypermethylation of the activity-dependent *BDNF* exon IV promoter region in the PFC, which leads to decreased exon IV expression (Roth et al., 2009). A follow-up study demonstrated additional methylation changes in the hippocampus and amygdala upon exposure to this stress paradigm. These effects were sex and brain region specific (hypermethylation of exon I in males in ventral hippocampus and of exon I in basolateral amygdala (BLA) in females) (Roth et al., 2014). These results were obtained using adult rats and highlight the robust methylation alterations in *BDNF* that occur through ELS. Finally, another study showed that communal nesting of the pups increased histone acetylation at the *BDNF* exon IV promoter (Branchi et al., 2011).

### ELS effects on synaptic plasticity and behavior

Irrespective of the MS paradigm, neurogenesis in the subventricular zone of the hippocampus appears to be consistently increased in the early postweaning phase and decreased during late adulthood. Moreover, synaptic plasticity related proteins such as neural cell adhesion molecule 1 (NCAM1) and synaptophysin are downregulated during adulthood after MS. Finally, a behavioral phenotype occurs in association with the temporal appearance of the above-mentioned changes, including memory impairment, learned helplessness, reduced social interaction, anhedonia and anxiety.

### Synthesis

These studies indicate that ELS induced alterations of BDNF expression in a brain-region specific and age-dependent manner and provide evidence that BDNF upregulation potentially acts as a neuroprotective mechanism upon ELS exposure.

## Interplay between BDNF and GC

### Effects of BDNF and GC on GR transcriptome

The GC- and BDNF-signaling pathways influenced by ELS are interlinked throughout life. A recent study confirmed that the GR-specific transcriptome is significantly altered by BDNF. Furthermore, simultaneous treatment of primary rat hippocampal neurons with a synthetic GC, dexamethasone (DEX), and BDNF induces the expression of a unique set of GC-BDNF responsive genes. The majority of these genes are involved in neurite outgrowth and differentiation (Lambert et al., 2013). In the same study, the authors established that BDNF leads to specific phosphorylation of the GR at serines 155 and 287 (Lambert et al., 2013). The latter serine (S287) is stress-hormone responsive, since DEX alone is sufficient to increase phosphorylation. In addition, increased S287 phosphorylation is observed in corticotropin-releasing hormone (CRH) expressing neurons in the parventricular nucleus (PVN) in mice that were exposed to 10 min of forced swim test.

### Impact of BDNF and GCs on brain morphology

It is well established that chronic stress affects the morphology of brain structures such as the hippocampus and the amygdala (Watanabe et al., 1992; Magarinos et al., 1996; Vyas et al., 2002). However, questions remain as how stress load and duration affect these brain regions on a structural level. Interestingly, a single exposure to emotional stress has been shown to increase dendritic length and number in amygdala and vice versa in the hippocampus (Rao et al., 2012). However, a recent study suggests that these neuronal phenotypes exclusively occur in rats displaying a vulnerable phenotype, with the degree of cytoarchitectural change predicting the changes in behavioral patterns (Cohen et al., 2014). Based on these findings it is of interest to understand if these stress effects on brain morphology are mediated by GCs. Corticosterone injections over the course of 3 days led to increased spine formation and concomitant spine elimination. In contrast to these findings, administering daily corticosterone over 10 days caused higher spine elimination (12.1–22.7%), but no increase in spine formation (Liston and Gan, 2011). Interestingly, the developing brain (P30) was even more sensitive. This GC effect seems to be mediated in the CNS directly and preferentially through MR. In another study investigating pubertal rats revealed that a single corticosterone administration evokes differential spatio-temporal effects in the PFC and the BLA (Kim et al., 2014). In particular, 6 days after a moderate dose of corticosterone injection (10 mg/kg) in the medial PFC the dendritic branches and lengths were decreased in parallel with working memory performance. Those effects returned to baseline 1 week after these measurements (day 12). In the BLA the effects of an acute corticosterone injection were slower in onset (day 12 after injection) and were also normalized after a week (day 20). When stress and acute corticosterone administration coincide, they antagonize each other rather than acting in an additive manner (Rao et al., 2012; Cohen et al., 2014).

GC and neurotrophin systems both act in antagonistic as well as in synergistic manners. BDNF and GC are involved in dendritic arborization, whereas BDNF is generally more associated with spine formation and stabilization with GC rather playing an important role in spine turnover (Jeanneteau and Chao, 2013). Mice carrying the minor allele (Met) of the human BDNF Val66Met (rs6265) variant, which alters the structural conformation of the BDNF pro-domain, display less branching in the dentate gyrus (Chen et al., 2006). With regard to GC, one study showed that chronic GC application results in spine loss in the barrel cortex. Interestingly, transient increased GC levels mostly affected newly formed spines, whereas chronically increased GCs affected spines that have been developed early in life (Liston and Gan, 2011).

### Molecular mechanisms of BDNF and GC interplay

**Figure 1:**
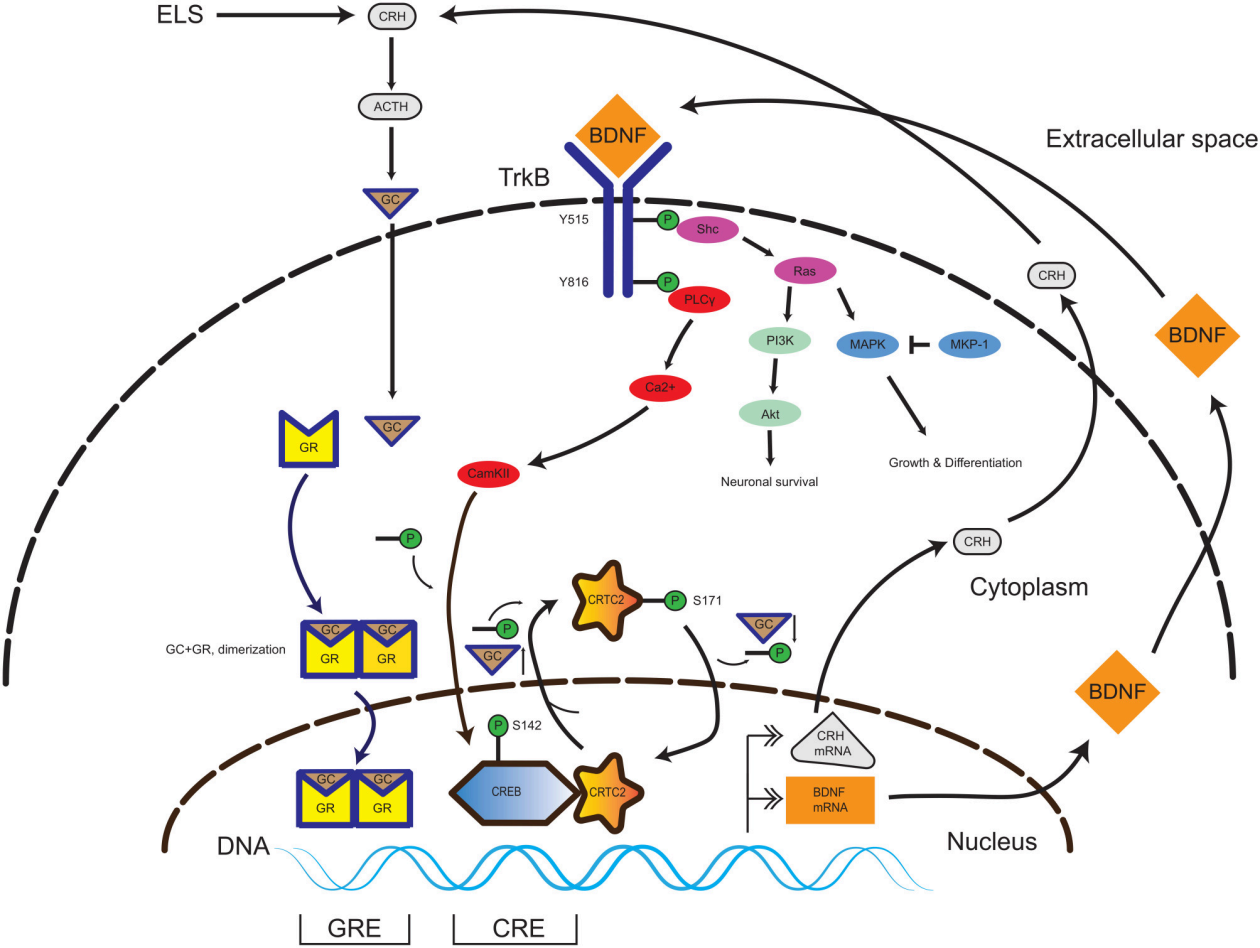
**Interplay of TrkB and GR signaling pathways in the CNS**. In the presence of BDNF, the TrkB receptor homodimerizes and initiates several signaling pathways promoting neuronal survival, growth and differentiation (Akt and MAPK). Activation of the PLCy pathway leads to CAMKII-mediated phosphorylation of the transcription factor CREB. In presence of a low amount of GC, the CREB-coactivator CRTC2 is dephosphorylated and translocates to the nucleus and binds to phospho-CREB. The phospho-CREB-CRTC2 complex binds at the CRH promoter and drives basal CRH expression in the PVN. Upon occurrence of ELS, the HPA axis signaling pathway is activated, yielding increasing GC levels. GC pass the plasma membrane and enter in to the cytosol and binds to GR, thereby inducing homodimerization (GR-GC complex). The GR-GC complex targets the BDNF promoter and drives basal BDNF production. Exceeding GC levels evoke a translocation of the CREB-coactivator CRTC2 to the cytosol and its phosphorylation, thereby inactivating CREB-dependent CRH production. Thus, the GR and TrkB pathways are calibrated and a specific balance of both GC and BDNF levels is necessary during neurodevelopment to keep homeostasis. Abbreviations: CNS, central nervous system; TrkB, tyrosine kinase receptor type 2; GR, glucocorticoid receptor; CAMKII, Calcium/Calmodulin-Dependent Protein Kinase II; CREB, cAMP Responsive Element Binding Protein; CRTC2, CREB Regulated Transcription Coactivator 2; ELS, early life stress; GC, glucocorticoids; BDNF, brain-derived neurotrophic factor; ACTH, adrenocorticotropic hormone; HPA axis, hypothalamus-pituitary-adrenal gland axis.

BDNF can directly influence the HPA-axis regulation through alterations of CRH expression levels. In primary hippocampal neurons, BDNF administration induced a three-fold increase in CRH expression. On the other hand, DEX administration led to repression of CRH, which could not be normalized by BDNF treatment (Jeanneteau et al., 2012). A chromatin immunoprecipitation experiment revealed that DEX treatment evoked increased GR-binding to the CRH promoter (Miller et al., 2011; Jeanneteau et al., 2012). In contrast to DEX, BDNF leads to an increase of cAMP response element-binding protein (CREB) -binding to its site on the CRH promoter, which is in proximity (22 bp) to the GR-binding site. The central mechanistic element in CRH regulation is the recruitment of CREB to the CRH promoter. For transcriptional activity, CREB requires the interaction with a coactivator protein named CREB-regulated transcription coactivator 2 (CRTC2). The increase of GC levels lead to the relocalization of the nuclear CRTC2 to the cytosol and thus decreased CREB transcriptional activity at the CRH promoter (Jeanneteau et al., 2012). In the same study, hypomorphic GR mice had increased BDNF expression and TrkB phosphorylation levels in the PVN in comparison to control littermates. This data is consistent with cross-talk between the neurotrophin and HPA-axis systems through the converging pathways, which are yet to be fully elucidated (Figure 1).

Another link between the GC- and BDNF-signaling pathways seems to involve the mitogen-activated protein kinase (MAPK) pathway. Chronic stress not only produces high levels of corticosterone and depressive-like behavior (de Kloet et al., 2005a), but also increases levels of a phosphatase in the MAPK pathway, (i.e., dual specificity phosphatase 1; MKP-1) in the brain. Chronic overexpression of MKP-1 induces detrimental effects by inhibiting axonal growth (Duric et al., 2010). Normalizing GC levels and consecutively MKP-1 expression levels leads to a restoration of stress-related depressive phenotypes through normalization of BDNF expression. Alternatively, constitutive knockdown of MPK-1 is associated with stress-resilience (Jeanneteau et al., 2010).

High GR levels decrease the abundance of the activity-dependent *BDNF* exon IV transcript in the dentate gyrus, CA1 and CA3 regions of the hippocampus without influencing exon I and II transcripts (Smith et al., 1995; Hansson et al., 2006). This calibration effect is corroborated by observations from adrenalectomized mice, in which corticosterone production is abolished and BDNF expression is increased in the CA1, CA3 and dentate gyrus of the hippocampus (Chao et al., 1998). In a further study it was demonstrated that acute GC activity evokes transiently increased tissue-plasminogen activator (tPA) protein levels. The presence of higher levels of tPA yields an increased proteolytic cleavage of pro-BDNF to mature BDNF. The higher amount of mature BDNF itself binds TrkB and enhances downstream MAPK phosphorylation, which is necessary for the formation of enhanced contextual fear memory (Revest et al., 2014).

## Conclusion

There is growing body of evidence that the GC–BDNF crosstalk is essential for the early-life programming of the HPA-axis and neurotrophin signaling. During early life, high BDNF and low GC levels are required for neuronal maintenance, synaptic integrity and dendritic spine stabilization in the hippocampus. BDNF-GC equilibrium is crucial throughout life as a major mechanism for stress response regulation. ELS can influence the set point of this equilibrium and thus cause long-term sensitizing effects on stress vulnerability. There is a body of evidence that ELS shifts BDNF as well as GR expression levels in the developing CNS. The long term effect of ELS exposure is a downregulation of BDNF expression (Table 1) and GR expression in the hippocampus (Sutanto et al., 1996; Aisa et al., 2007). The combination of both low BDNF and low GR expression favors the vulnerability to develop stress-related disorders during adolescence and adulthood, especially upon additional stress exposures. Phenotypes associated with the ELS-induced reductions of BDNF and GR expression in rodents (Table 1) have been additionally observed to be associated with the interaction of the BDNF Met risk allele (Val66Met) and childhood trauma in humans (Molendijk et al., 2012; Aas et al., 2013). Therefore, it is important to understand that genetic and epigenetic factors moderate the long term consequences of early adversity (Daskalakis and Binder, 2015). From a therapeutic point of view, preventing steep GC elevations induced by ELS has beneficial effects through constitutive BDNF expression with a concomitant stable, physiological calibration of the GC- and BDNF-signaling pathways.

### Conflict of interest statement

The authors declare that the research was conducted in the absence of any commercial or financial relationships that could be construed as a potential conflict of interest.

## References

[B1] Aas M. Haukvik U. K. Djurovic S. Bergmann O. Athanasiu L. Tesli M. S.. (2013). BDNF val66met modulates the association between childhood trauma, cognitive and brain abnormalities in psychoses. Prog. Neuropsychopharmacol. Biol. Psychiatry 46, 181–188. 10.1016/j.pnpbp.2013.07.008 23876786

[B2] Aisa B. Elizalde N. Tordera R. Lasheras B. Del Rio J. Ramirez M. J. (2009). Effects of neonatal stress on markers of synaptic plasticity in the hippocampus: implications for spatial memory. Hippocampus 19, 1222–1231. 10.1002/hipo.20586 19309038

[B3] Aisa B. Tordera R. Lasheras B. Del Rio J. Ramirez M. J. (2007). Cognitive impairment associated to HPA axis hyperactivity after maternal separation in rats. Psychoneuroendocrinology 32, 256–266. 10.1016/j.psyneuen.2006.12.013 17307298

[B4] Alleva E. Francia N. (2009). Psychiatric vulnerability: suggestions from animal models and role of neurotrophins. Neurosci. Biobehav. Rev. 33, 525–536. 10.1016/j.neubiorev.2008.09.004 18824030

[B5] Biggio F. Pisu M. G. Garau A. Boero G. Locci V. Mostallino M. C.. (2014). Maternal separation attenuates the effect of adolescent social isolation on HPA axis responsiveness in adult rats. Eur. Neuropsychopharmacol. 24, 1152–1161. 10.1016/j.euroneuro.2014.03.009 24745548

[B6] Branchi I. Karpova N. N. D'andrea I. Castrén E. Alleva E. (2011). Epigenetic modifications induced by early enrichment are associated with changes in timing of induction of BDNF expression. Neurosci. Lett. 495, 168–172. 10.1016/j.neulet.2011.03.038 21420470

[B7] Bremner J. D. Vythilingam M. Vermetten E. Southwick S. M. McGlashan T. Nazeer A.. (2003). MRI and PET study of deficits in hippocampal structure and function in women with childhood sexual abuse and posttraumatic stress disorder. Am. J. Psychiatry 160, 924–932. 10.1176/appi.ajp.160.5.924 12727697

[B8] Calabrese F. van der Doelen R. H. Guidotti G. Racagni G. Kozicz T. Homberg J. R.. (2015). Exposure to early life stress regulates Bdnf expression in SERT mutant rats in an anatomically selective fashion. J. Neurochem. 132, 146–154. 10.1111/jnc.12846 25087780

[B9] Champagne D. L. Bagot R. C. van Hasselt F. Ramakers G. Meaney M. J. de Kloet E. R.. (2008). Maternal care and hippocampal plasticity: evidence for experience-dependent structural plasticity, altered synaptic functioning, and differential responsiveness to glucocorticoids and stress. J. Neurosci. 28, 6037–6045. 10.1523/JNEUROSCI.0526-08.2008 18524909 PMC6670331

[B10] Chao H. M. Sakai R. R. Ma L. Y. McEwen B. S. (1998). Adrenal steroid regulation of neurotrophic factor expression in the rat hippocampus. Endocrinology 139, 3112–3118. 10.1210/endo.139.7.6114 9645683

[B11] Chao M. V. (2003). Neurotrophins and their receptors: a convergence point for many signalling pathways. Nat. Rev. Neurosci. 4, 299–309. 10.1038/nrn1078 12671646

[B12] Chen Z. Y. Jing D. Bath K. G. Ieraci A. Khan T. Siao C. J.. (2006). Genetic variant BDNF (Val66Met) polymorphism alters anxiety-related behavior. Science 314, 140–143. 10.1126/science.1129663 17023662 PMC1880880

[B13] Choy K. H. de Visser Y. Nichols N. R. van den Buuse M. (2008). Combined neonatal stress and young-adult glucocorticoid stimulation in rats reduce BDNF expression in hippocampus: effects on learning and memory. Hippocampus 18, 655–667. 10.1002/hipo.20425 18398848

[B14] Cirulli F. Reif A. Herterich S. Lesch K. P. Berry A. Francia N.. (2011). A novel BDNF polymorphism affects plasma protein levels in interaction with early adversity in rhesus macaques. Psychoneuroendocrinology 36, 372–379. 10.1016/j.psyneuen.2010.10.019 21145664 PMC3046296

[B15] Cohen H. Kozlovsky N. Matar M. A. Zohar J. Kaplan Z. (2014). Distinctive hippocampal and amygdalar cytoarchitectural changes underlie specific patterns of behavioral disruption following stress exposure in an animal model of PTSD. Eur. Neuropsychopharmacol. 24, 1925–1944. 10.1016/j.euroneuro.2014.09.009 25451698

[B16] Daskalakis N. P. Bagot R. C. Parker K. J. Vinkers C. H. de Kloet E. R. (2013). The three-hit concept of vulnerability and resilience: toward understanding adaptation to early-life adversity outcome. Psychoneuroendocrinology 38, 1858–1873. 10.1016/j.psyneuen.2013.06.008 23838101 PMC3773020

[B17] Daskalakis N. P. Binder E. B. (2015). Schizophrenia in the spectrum of gene-stress interactions: the FKBP5 example. Schizophr. Bull. 41, 323–329. 10.1093/schbul/sbu189 25592294 PMC4332957

[B18] Daskalakis N. P. Claessens S. E. Laboyrie J. J. Enthoven L. Oitzl M. S. Champagne D. L.. (2011). The newborn rat's stress system readily habituates to repeated and prolonged maternal separation, while continuing to respond to stressors in context dependent fashion. Horm. Behav. 60, 165–176. 10.1016/j.yhbeh.2011.04.003 21570400

[B19] Daskalakis N. P. Diamantopoulou A. Claessens S. E. Remmers E. Tjalve M. Oitzl M. S.. (2014). Early experience of a novel-environment in isolation primes a fearful phenotype characterized by persistent amygdala activation. Psychoneuroendocrinology 39, 39–57. 10.1016/j.psyneuen.2013.09.021 24275003

[B20] Daskalakis N. P. Oitzl M. S. Schachinger H. Champagne D. L. de Kloet E. R. (2012). Testing the cumulative stress and mismatch hypotheses of psychopathology in a rat model of early-life adversity. Physiol. Behav. 106, 707–721. 10.1016/j.physbeh.2012.01.015 22306534

[B21] Daskalakis N. P. Yehuda R. (2014). Site-specific methylation changes in the glucocorticoid receptor exon 1F promoter in relation to life adversity: systematic review of contributing factors. Front. Neurosci. 8:369. 10.3389/fnins.2014.00369 25484853 PMC4240065

[B22] de Kloet E. R. (2003). Hormones, brain and stress. Endocr. Regul. 37, 51–68. 12932191

[B23] de Kloet E. R. Joëls M. Holsboer F. (2005a). Stress and the brain: from adaptation to disease. Nat. Rev. Neurosci. 6, 463–475. 10.1038/nrn1683 15891777

[B24] de Kloet E. R. Sibug R. M. Helmerhorst F. M. Schmidt M. V. (2005b). Stress, genes and the mechanism of programming the brain for later life. Neurosci. Biobehav. Rev. 29, 271–281. 10.1016/j.neubiorev.2004.10.008 15811498

[B25] De Kloet E. R. Vreugdenhil E. Oitzl M. S. Joels M. (1998). Brain corticosteroid receptor balance in health and disease. Endocr. Rev. 19, 269–301. 10.1210/er.19.3.269 9626555

[B26] Dimatelis J. J. Russell V. A. Stein D. J. Daniels W. M. (2014). Methamphetamine reversed maternal separation-induced decrease in nerve growth factor in the ventral hippocampus. Metab. Brain Dis. 29, 433–439. 10.1007/s11011-014-9481-z 24407463

[B27] Dimatelis J. J. Stein D. J. Russell V. A. (2012). Behavioral changes after maternal separation are reversed by chronic constant light treatment. Brain Res. 1480, 61–71. 10.1016/j.brainres.2012.07.013 22975437

[B28] Duan W. Lee J. Guo Z. Mattson M. P. (2001). Dietary restriction stimulates BDNF production in the brain and thereby protects neurons against excitotoxic injury. J. Mol. Neurosci. 16, 1–12. 10.1385/JMN:16:1:1 11345515

[B29] Duric V. Banasr M. Licznerski P. Schmidt H. D. Stockmeier C. A. Simen A. A.. (2010). A negative regulator of MAP kinase causes depressive behavior. Nat. Med. 16, 1328–1332. 10.1038/nm.2219 20953200 PMC3066515

[B30] Elzinga B. M. Molendijk M. L. Oude Voshaar R. C. Bus B. A. Prickaerts J. Spinhoven P.. (2011). The impact of childhood abuse and recent stress on serum brain-derived neurotrophic factor and the moderating role of BDNF Val66Met. Psychopharmacology 214, 319–328. 10.1007/s00213-010-1961-1 20703451 PMC3045516

[B31] Enthoven L. Schmidt M. V. Cheung Y. H. van der Mark M. H. de Kloet E. R. Oitzl M. S. (2010). Ontogeny of the HPA axis of the CD1 mouse following 24 h maternal deprivation at pnd 3. Int. J. Dev. Neurosci. 28, 217–224. 10.1016/j.ijdevneu.2009.10.006 19897026

[B32] Faure J. Uys J. D. Marais L. Stein D. J. Daniels W. M. (2006). Early maternal separation followed by later stressors leads to dysregulation of the HPA-axis and increases in hippocampal NGF and NT-3 levels in a rat model. Metab. Brain Dis. 21, 181–188. 10.1007/s11011-006-9013-6 16850259

[B33] Faure J. Uys J. D. Marais L. Stein D. J. Daniels W. M. (2007). Early maternal separation alters the response to traumatization: resulting in increased levels of hippocampal neurotrophic factors. Metab. Brain Dis. 22, 183–195. 10.1007/s11011-007-9048-3 17468977

[B34] Ghosh A. Greenberg M. E. (1995). Distinct roles for bFGF and NT-3 in the regulation of cortical neurogenesis. Neuron 15, 89–103. 10.1016/0896-6273(95)90067-5 7619533

[B35] Grassi-Oliveira R. Stein L. M. Lopes R. P. Teixeira A. L. Bauer M. E. (2008). Low plasma brain-derived neurotrophic factor and childhood physical neglect are associated with verbal memory impairment in major depression–a preliminary report. Biol. Psychiatry 64, 281–285. 10.1016/j.biopsych.2008.02.023 18406398

[B36] Greisen M. H. Altar C. A. Bolwig T. G. Whitehead R. Wortwein G. (2005). Increased adult hippocampal brain-derived neurotrophic factor and normal levels of neurogenesis in maternal separation rats. J. Neurosci. Res. 79, 772–778. 10.1002/jnr.20418 15690366

[B37] Gulemetova R. Drolet G. Kinkead R. (2013). Neonatal stress augments the hypoxic chemoreflex of adult male rats by increasing AMPA receptor-mediated modulation. Exp. Physiol. 98, 1312–1324. 10.1113/expphysiol.2013.072090 23603375

[B38] Gunnar M. Quevedo K. (2007). The neurobiology of stress and development. Annu. Rev. Psychol. 58, 145–173. 10.1146/annurev.psych.58.110405.085605 16903808

[B39] Hansson A. C. Sommer W. H. Metsis M. Stromberg I. Agnati L. F. Fuxe K. (2006). Corticosterone actions on the hippocampal brain-derived neurotrophic factor expression are mediated by exon IV promoter. J. Neuroendocrinol. 18, 104–114. 10.1111/j.1365-2826.2005.01390.x 16420279

[B40] Hill R. A. Kiss Von Soly S. Ratnayake U. Klug M. Binder M. D. Hannan A. J.. (2014a). Long-term effects of combined neonatal and adolescent stress on brain-derived neurotrophic factor and dopamine receptor expression in the rat forebrain. Biochim. Biophys. Acta 1842, 2126–2135. 10.1016/j.bbadis.2014.08.009 25159716

[B41] Hill R. A. Klug M. Kiss Von Soly S. Binder M. D. Hannan A. J. van den Buuse M. (2014b). Sex-specific disruptions in spatial memory and anhedonia in a “two hit” rat model correspond with alterations in hippocampal brain-derived neurotrophic factor expression and signaling. Hippocampus 24, 1197–1211. 10.1002/hipo.22302 24802968

[B42] Ivy A. S. Brunson K. L. Sandman C. Baram T. Z. (2008). Dysfunctional nurturing behavior in rat dams with limited access to nesting material: a clinically relevant model for early-life stress. Neuroscience 154, 1132–1142. 10.1016/j.neuroscience.2008.04.019 18501521 PMC2517119

[B43] Jeanneteau F. Chao M. V. (2013). Are BDNF and glucocorticoid activities calibrated? Neuroscience 239, 173–195. 10.1016/j.neuroscience.2012.09.017 23022538 PMC3581703

[B44] Jeanneteau F. Deinhardt K. Miyoshi G. Bennett A. M. Chao M. V. (2010). The MAP kinase phosphatase MKP-1 regulates BDNF-induced axon branching. Nat. Neurosci. 13, 1373–1379. 10.1038/nn.2655 20935641 PMC2971689

[B45] Jeanneteau F. D. Lambert W. M. Ismaili N. Bath K. G. Lee F. S. Garabedian M. J.. (2012). BDNF and glucocorticoids regulate corticotrophin-releasing hormone (CRH) homeostasis in the hypothalamus. Proc. Natl. Acad. Sci. U.S.A. 109, 1305–1310. 10.1073/pnas.1114122109 22232675 PMC3268297

[B46] Kim H. Yi J. H. Choi K. Hong S. Shin K. S. Kang S. J. (2014). Regional differences in acute corticosterone-induced dendritic remodeling in the rat brain and their behavioral consequences. BMC Neurosci. 15:65. 10.1186/1471-2202-15-65 24884833 PMC4038707

[B47] Klengel T. Mehta D. Anacker C. Rex-Haffner M. Pruessner J. C. Pariante C. M.. (2013). Allele-specific FKBP5 DNA demethylation mediates gene-childhood trauma interactions. Nat. Neurosci. 16, 33–41. 10.1038/nn.3275 23201972 PMC4136922

[B48] Kranz T. M. Goetz R. R. Walsh-Messinger J. Goetz D. Antonius D. Dolgalev I.. (2015a). Rare variants in the neurotrophin signaling pathway implicated in schizophrenia risk. Schizophr. Res. 168, 421–428. 10.1016/j.schres.2015.07.002 26215504 PMC4591185

[B49] Kranz T. M. Harroch S. Manor O. Lichtenberg P. Friedlander Y. Seandel M.. (2015b). De novo mutations from sporadic schizophrenia cases highlight important signaling genes in an independent sample. Schizophr. Res. 166, 119–124. 10.1016/j.schres.2015.05.042 26091878 PMC4512856

[B50] Kuma H. Miki T. Matsumoto Y. Gu H. Li H. P. Kusaka T.. (2004). Early maternal deprivation induces alterations in brain-derived neurotrophic factor expression in the developing rat hippocampus. Neurosci. Lett. 372, 68–73. 10.1016/j.neulet.2004.09.012 15531090

[B51] Kundakovic M. Lim S. Gudsnuk K. Champagne F. A. (2013). Sex-specific and strain-dependent effects of early life adversity on behavioral and epigenetic outcomes. Front. Psychiatry 4:78. 10.3389/fpsyt.2013.00078 23914177 PMC3730082

[B52] Lambert W. M. Xu C. F. Neubert T. A. Chao M. V. Garabedian M. J. Jeanneteau F. D. (2013). Brain-derived neurotrophic factor signaling rewrites the glucocorticoid transcriptome via glucocorticoid receptor phosphorylation. Mol. Cell. Biol. 33, 3700–3714. 10.1128/MCB.00150-13 23878391 PMC3753865

[B53] Lee J. Duan W. Mattson M. P. (2002). Evidence that brain-derived neurotrophic factor is required for basal neurogenesis and mediates, in part, the enhancement of neurogenesis by dietary restriction in the hippocampus of adult mice. J. Neurochem. 82, 1367–1375. 10.1046/j.1471-4159.2002.01085.x 12354284

[B54] Lee K. Y. Miki T. Yokoyama T. Ueki M. Warita K. Suzuki S.. (2012). Neonatal repetitive maternal separation causes long-lasting alterations in various neurotrophic factor expression in the cerebral cortex of rats. Life Sci. 90, 578–584. 10.1016/j.lfs.2012.01.021 22365961

[B55] Lippmann M. Bress A. Nemeroff C. B. Plotsky P. M. Monteggia L. M. (2007). Long-term behavioural and molecular alterations associated with maternal separation in rats. Eur. J. Neurosci. 25, 3091–3098. 10.1111/j.1460-9568.2007.05522.x 17561822

[B56] Liston C. Gan W.-B. (2011). Glucocorticoids are critical regulators of dendritic spine development and plasticity *in vivo*. Proc. Natl. Acad. Sci. U.S.A. 108, 16074–16079. 10.1073/pnas.1110444108 21911374 PMC3179117

[B57] Liu D. Diorio J. Day J. C. Francis D. D. Meaney M. J. (2000). Maternal care, hippocampal synaptogenesis and cognitive development in rats. Nat. Neurosci. 3, 799–806. 10.1038/77702 10903573

[B58] Liu D. Diorio J. Tannenbaum B. Caldji C. Francis D. Freedman A.. (1997). Maternal care, hippocampal glucocorticoid receptors, and hypothalamic-pituitary-adrenal responses to stress. Science 277, 1659–1662. 10.1126/science.277.5332.1659 9287218

[B59] Llorente R. Miguel-Blanco C. Aisa B. Lachize S. Borcel E. Meijer O. C.. (2011). Long term sex-dependent psychoneuroendocrine effects of maternal deprivation and juvenile unpredictable stress in rats. J. Neuroendocrinol. 23, 329–344. 10.1111/j.1365-2826.2011.02109.x 21219484

[B60] MacQueen G. M. Ramakrishnan K. Ratnasingan R. Chen B. Young L. T. (2003). Desipramine treatment reduces the long-term behavioural and neurochemical sequelae of early-life maternal separation. Int. J. Neuropsychopharmacol. 6, 391–396. 10.1017/S1461145703003729 14641986

[B61] Magarinos A. M. McEwen B. S. Flugge G. Fuchs E. (1996). Chronic psychosocial stress causes apical dendritic atrophy of hippocampal CA3 pyramidal neurons in subordinate tree shrews. J. Neurosci. 16, 3534–3540. 8627386 10.1523/JNEUROSCI.16-10-03534.1996 PMC6579123

[B62] McEwen B. S. (1998). Protective and damaging effects of stress mediators. N. Engl. J. Med. 338, 171–179. 10.1056/NEJM199801153380307 9428819

[B63] McGowan P. O. Sasaki A. D'Alessio A. C. Dymov S. Labonté B. Szyf M.. (2009). Epigenetic regulation of the glucocorticoid receptor in human brain associates with childhood abuse. Nat. Neurosci. 12, 342–348. 10.1038/nn.2270 19234457 PMC2944040

[B64] Miki T. Lee K. Y. Yokoyama T. Liu J. Q. Kusaka T. Suzuki S.. (2013). Differential effects of neonatal maternal separation on the expression of neurotrophic factors in rat brain. II: regional differences in the cerebellum versus the cerebral cortex. Okajimas Folia Anat. Jpn. 90, 53–58. 10.2535/ofaj.90 24670490

[B65] Miki T. Yokoyama T. Kusaka T. Suzuki S. Ohta K. Warita K.. (2014). Early postnatal repeated maternal deprivation causes a transient increase in OMpg and BDNF in rat cerebellum suggesting precocious myelination. J. Neurol. Sci. 336, 62–67. 10.1016/j.jns.2013.10.007 24157309

[B66] Miller L. Foradori C. D. Lalmansingh A. S. Sharma D. Handa R. J. Uht R. M. (2011). Histone deacetylase 1 (HDAC1) participates in the down-regulation of corticotropin releasing hormone gene (crh) expression. Physiol. Behav. 104, 312–320. 10.1016/j.physbeh.2011.03.026 21463644 PMC3650854

[B67] Molendijk M. L. van Tol M. J. Penninx B. W. van der Wee N. J. Aleman A. Veltman D. J.. (2012). BDNF val66met affects hippocampal volume and emotion-related hippocampal memory activity. Transl. Psychiatry 2, e74. 10.1038/tp.2011.72 22832736 PMC3309548

[B68] Murgatroyd C. Patchev A. V. Wu Y. Micale V. Bockmühl Y. Fischer D.. (2009). Dynamic DNA methylation programs persistent adverse effects of early-life stress. Nat. Neurosci. 12, 1559–1566. 10.1038/nn.2436 19898468

[B69] Nair A. Vadodaria K. C. Banerjee S. B. Benekareddy M. Dias B. G. Duman R. S.. (2007). Stressor-specific regulation of distinct brain-derived neurotrophic factor transcripts and cyclic AMP response element-binding protein expression in the postnatal and adult rat hippocampus. Neuropsychopharmacology 32, 1504–1519. 10.1038/sj.npp.1301276 17164818

[B70] Ninan I. Bath K. G. Dagar K. Perez-Castro R. Plummer M. R. Lee F. S.. (2010). The BDNF Val66Met polymorphism impairs NMDA receptor-dependent synaptic plasticity in the hippocampus. J. Neurosci. 30, 8866–8870. 10.1523/JNEUROSCI.1405-10.2010 20592208 PMC2911131

[B71] Ognibene E. Adriani W. Caprioli A. Ghirardi O. Ali S. F. Aloe L.. (2008). The effect of early maternal separation on brain derived neurotrophic factor and monoamine levels in adult heterozygous reeler mice. Prog. Neuropsychopharmacol. Biol. Psychiatry 32, 1269–1276. 10.1016/j.pnpbp.2008.03.023 18501492

[B72] Pang P. T. Teng H. K. Zaitsev E. Woo N. T. Sakata K. Zhen S.. (2004). Cleavage of proBDNF by tPA/plasmin is essential for long-term hippocampal plasticity. Science 306, 487–491. 10.1126/science.1100135 15486301

[B73] Pattwell S. S. Bath K. G. Perez-Castro R. Lee F. S. Chao M. V. Ninan I. (2012). The BDNF Val66Met polymorphism impairs synaptic transmission and plasticity in the infralimbic medial prefrontal cortex. J. Neurosci. 32, 2410–2421. 10.1523/JNEUROSCI.5205-11.2012 22396415 PMC3532006

[B74] Pinheiro R. M. de Lima M. N. Portal B. C. Busato S. B. Falavigna L. Ferreira R. D.. (2015). Long-lasting recognition memory impairment and alterations in brain levels of cytokines and BDNF induced by maternal deprivation: effects of valproic acid and topiramate. J. Neural Transm. 122, 709–719. 10.1007/s00702-014-1303-2 25182413

[B75] Rao R. P. Anilkumar S. McEwen B. S. Chattarji S. (2012). Glucocorticoids protect against the delayed behavioral and cellular effects of acute stress on the amygdala. Biol. Psychiatry 72, 466–475. 10.1016/j.biopsych.2012.04.008 22572034 PMC3753225

[B76] Revest J. M. Le Roux A. Roullot-Lacarrière V. Kaouane N. Vallée M. Kasanetz F.. (2014). BDNF-TrkB signaling through Erk1/2 MAPK phosphorylation mediates the enhancement of fear memory induced by glucocorticoids. Mol. Psychiatry 19, 1001–1009. 10.1038/mp.2013.134 24126929 PMC4195976

[B77] Rice C. J. Sandman C. A. Lenjavi M. R. Baram T. Z. (2008). A novel mouse model for acute and long-lasting consequences of early life stress. Endocrinology 149, 4892–4900. 10.1210/en.2008-0633 18566122 PMC2582918

[B78] Roceri M. Cirulli F. Pessina C. Peretto P. Racagni G. Riva M. A. (2004). Postnatal repeated maternal deprivation produces age-dependent changes of brain-derived neurotrophic factor expression in selected rat brain regions. Biol. Psychiatry 55, 708–714. 10.1016/j.biopsych.2003.12.011 15038999

[B79] Roceri M. Hendriks W. Racagni G. Ellenbroek B. A. Riva M. A. (2002). Early maternal deprivation reduces the expression of BDNF and NMDA receptor subunits in rat hippocampus. Mol. Psychiatry 7, 609–616. 10.1038/sj.mp.4001036 12140784

[B80] Rosenfeld P. Wetmore J. B. Levine S. (1992). Effects of repeated maternal separations on the adrenocortical response to stress of preweanling rats. Physiol. Behav. 52, 787–791. 10.1016/0031-9384(92)90415-X 1409954

[B81] Roth T. L. Lubin F. D. Funk A. J. Sweatt J. D. (2009). Lasting epigenetic influence of early-life adversity on the BDNF gene. Biol. Psychiatry 65, 760–769. 10.1016/j.biopsych.2008.11.028 19150054 PMC3056389

[B82] Roth T. L. Matt S. Chen K. Blaze J. (2014). Bdnf DNA methylation modifications in the hippocampus and amygdala of male and female rats exposed to different caregiving environments outside the homecage. Dev. Psychobiol. 56, 1755–1763. 10.1002/dev.21218 24752649 PMC4205217

[B83] Ruby E. Rothman K. Corcoran C. Goetz R. R. Malaspina D. (2015). Influence of early trauma on features of schizophrenia. Early Interv. Psychiatry. 10.1111/eip.12239. [Epub ahead of print]. 25808607 PMC4580512

[B84] Sapolsky R. M. Meaney M. J. (1986). Maturation of the adrenocortical stress response: neuroendocrine control mechanisms and the stress hyporesponsive period. Brain Res. 396, 64–76. 10.1016/0165-0173(86)90010-x 3011218

[B85] Schaaf M. J. Workel J. O. Lesscher H. M. Vreugdenhil E. Oitzl M. S. de Kloet E. R. (2001). Correlation between hippocampal BDNF mRNA expression and memory performance in senescent rats. Brain Res. 915, 227–233. 10.1016/S0006-8993(01)02855-4 11595212

[B86] Smith M. A. Makino S. Kim S. Y. Kvetnansky R. (1995). Stress increases brain-derived neurotropic factor messenger ribonucleic acid in the hypothalamus and pituitary. Endocrinology 136, 3743–3750. 7649080 10.1210/endo.136.9.7649080

[B87] Solas M. Aisa B. Mugueta M. C. Del Rio J. Tordera R. M. Ramirez M. J. (2010). Interactions between age, stress and insulin on cognition: implications for Alzheimer's disease. Neuropsychopharmacology 35, 1664–1673. 10.1038/npp.2010.13 20182419 PMC3055481

[B88] Suri D. Veenit V. Sarkar A. Thiagarajan D. Kumar A. Nestler E. J.. (2013). Early stress evokes age-dependent biphasic changes in hippocampal neurogenesis, BDNF expression, and cognition. Biol. Psychiatry 73, 658–666. 10.1016/j.biopsych.2012.10.023 23237316 PMC4051354

[B89] Sutanto W. Rosenfeld P. de Kloet E. R. Levine S. (1996). Long-term effects of neonatal maternal deprivation and ACTH on hippocampal mineralocorticoid and glucocorticoid receptors. Brain Res. Dev. Brain Res. 92, 156–163. 10.1016/0165-3806(95)00213-8 8738122

[B90] van Oers H. J. de Kloet E. R. Whelan T. Levine S. (1998). Maternal deprivation effect on the infant's neural stress markers is reversed by tactile stimulation and feeding but not by suppressing corticosterone. J. Neurosci. 18, 10171–10179. 9822770 10.1523/JNEUROSCI.18-23-10171.1998 PMC6793306

[B91] Vyas A. Mitra R. Shankaranarayana Rao B. S. Chattarji S. (2002). Chronic stress induces contrasting patterns of dendritic remodeling in hippocampal and amygdaloid neurons. J. Neurosci. 22, 6810–6818. 12151561 10.1523/JNEUROSCI.22-15-06810.2002 PMC6758130

[B92] Watanabe Y. Gould E. McEwen B. S. (1992). Stress induces atrophy of apical dendrites of hippocampal CA3 pyramidal neurons. Brain Res. 588, 341–345. 10.1016/0006-8993(92)91597-8 1393587

[B93] Weaver I. C. Cervoni N. Champagne F. A. D'Alessio A. C. Sharma S. Seckl J. R.. (2004). Epigenetic programming by maternal behavior. Nat. Neurosci. 7, 847–854. 10.1038/nn1276 15220929

[B94] Xue X. Shao S. Wang W. Shao F. (2013). Maternal separation induces alterations in reversal learning and brain-derived neurotrophic factor expression in adult rats. Neuropsychobiology 68, 243–249. 10.1159/000356188 24280707

[B95] Yu H. Wang D. D. Wang Y. Liu T. Lee F. S. Chen Z. Y. (2012). Variant brain-derived neurotrophic factor Val66Met polymorphism alters vulnerability to stress and response to antidepressants. J. Neurosci. 32, 4092–4101. 10.1523/JNEUROSCI.5048-11.2012 22442074 PMC3319323

[B96] Zhang L. X. Levine S. Dent G. Zhan Y. Xing G. Okimoto D.. (2002). Maternal deprivation increases cell death in the infant rat brain. Brain Res. Dev. Brain Res. 133, 1–11. 10.1016/S0926-6410(01)00118-5 11850058

[B97] Zimmerberg B. Foote H. E. Van Kempen T. A. (2009). Olfactory association learning and brain-derived neurotrophic factor in an animal model of early deprivation. Dev. Psychobiol. 51, 333–344. 10.1002/dev.20373 19308959

